# Assessment of Treatment Response by Total Tumor Volume and Global Apparent Diffusion Coefficient Using Diffusion-Weighted MRI in Patients with Metastatic Bone Disease: A Feasibility Study

**DOI:** 10.1371/journal.pone.0091779

**Published:** 2014-04-07

**Authors:** Matthew D. Blackledge, David J. Collins, Nina Tunariu, Matthew R. Orton, Anwar R. Padhani, Martin O. Leach, Dow-Mu Koh

**Affiliations:** 1 Radiotherapy and Imaging Division, Cancer Research UK and Engineering and Physical Sciences Research Council Cancer Imaging Centre at The Institute of Cancer Research and The Royal Marsden National Health Service Foundation Trust, Sutton, United Kingdom; 2 Paul Strickland Scanner Centre, Mount Vernon Cancer Centre, Northwood, Middlesex, United Kingdom; University of Pécs Medical School, Hungary

## Abstract

We describe our semi-automatic segmentation of whole-body diffusion-weighted MRI (WBDWI) using a Markov random field (MRF) model to derive tumor total diffusion volume (tDV) and associated global apparent diffusion coefficient (gADC); and demonstrate the feasibility of using these indices for assessing tumor burden and response to treatment in patients with bone metastases.

WBDWI was performed on eleven patients diagnosed with bone metastases from breast and prostate cancers before and after anti-cancer therapies. Semi-automatic segmentation incorporating a MRF model was performed in all patients below the C4 vertebra by an experienced radiologist with over eight years of clinical experience in body DWI. Changes in tDV and gADC distributions were compared with overall response determined by all imaging, tumor markers and clinical findings at serial follow up. The segmentation technique was possible in all patients although erroneous volumes of interest were generated in one patient because of poor fat suppression in the pelvis, requiring manual correction. Responding patients showed a larger increase in gADC (median change = +0.18, range = −0.07 to +0.78×10^−3^ mm^2^/s) after treatment compared to non-responding patients (median change = −0.02, range = −0.10 to +0.05×10^−3^ mm^2^/s, p = 0.05, Mann-Whitney test), whereas non-responding patients showed a significantly larger increase in tDV (median change = +26%, range = +3 to +284%) compared to responding patients (median change = −50%, range = −85 to +27%, p = 0.02, Mann-Whitney test). Semi-automatic segmentation of WBDWI is feasible for metastatic bone disease in this pilot cohort of 11 patients, and could be used to quantify tumor total diffusion volume and median global ADC for assessing response to treatment.

## Introduction

The confident detection of metastatic bone disease and the assessment of treatment response of bone disease remains one of the major unfulfilled needs in oncology. However, recent studies using whole body diffusion-weighted MR imaging (WBDWI) have shown high diagnostic accuracy in detecting metastatic bone disease in patients with non-small cell lung cancer, malignant melanoma and breast cancers [1], [2]. There is also compelling evidence that the apparent diffusion coefficient derived from WBDWI provides a potential quantitative response biomarker that reflects tissue cellularity and has been shown to increase in responders to a range of anti-tumor treatments [3], [4].

WBDWI is a relatively recent imaging technique developed independently by Ballon and Takahara [5], [6]. It employs axial fat-suppressed diffusion-weighted MRI acquired at multiple anatomical stations using high diffusion sensitizing gradients (b-values). Due to the combination of the relatively low diffusion rates and high T_2_ relaxation time of water in tumor tissues, metastases typically appear hyper-intense compared with normal background tissue, thus providing excellent contrast for disease visualization. At high b-values (e.g. b = 800–1000 s/mm^2^), the sensitivity of WBDWI for detecting bone disease has been shown to be equivalent or better than skeletal scintigraphy (SS), but with superior spatial resolution to both SS and 18FDG-PET [7]–[9]. Further suppression of signal from healthy tissues may be achieved by applying the ‘computed Diffusion-Weighted MRI’ (cDWI) technique [10], which extrapolates images of higher b-value (assuming a monoexponential relationship between signal intensity and b-value). The resulting high lesion-to-background ratio facilitates segmentation of lesions to provide estimates of total tumor diffusion volumes (tDV) and the associated global median Apparent Diffusion Coefficient (gADC). By performing a similar segmentation after treatment, changes in tDV and gADC may be used to assess the treatment response of metastatic bone disease, thus providing two quantitative metrics from a single radiologic investigation.

Commercial software that enables radiologists to manually define or region-grow individual Volumes Of Interest (VOI) is beginning to emerge. However, in patients with metastatic bone disease, the number of metastases can be large, making it impractical to use such software to evaluate multiple lesions. Our approach employs a recently developed region-growing technique [11] to remove unwanted signal from background using cDWI and then applies an advanced thresholding algorithm (using a Markov random field model) to extract VOIs from multiple lesions across the body.

In this manuscript, we describe our semi-automatic segmentation of WBDWI to derive tumor diffusion volumes (tDV) and the associated global median ADC (gADC); and demonstrate the feasibility of using these indices for assessing tumor response to treatment in patients with bone metastases.

## Materials and Methods

### Ethics statement

Patients gave written informed consent and the Research Ethics Committee of the Royal Marsden Hospital approved the research study.

### Study population

Imaging from eleven consecutive patients with metastatic bone disease who underwent clinical WBDWI before and after chemotherapy was evaluated: four female patients with metastatic breast cancer (mean age = 51 years, range = 43–63 years) and seven male patients with metastatic prostate cancer (mean age = 64 years, range = 40–78 years). Further details of these patients including treatment type are shown in **Table 1**. The inclusion criteria were: (1) Patients with predominant metastatic bone disease demonstrated on CT, MRI, SS and/or ^18^FDG-PET. (2) Patients who were treatment naïve or showed recent disease progression, and were about to commence anti-tumor treatment. Patients with claustrophobia or contraindications to MRI examinations were not included in the study.

**Table 1:** Clinical details of the patient cohort included in the study.

Patient ID	Patient Diagnosis	Treatment administered*	Time between scans (weeks)	Overall treatment response
Patient 1	Breast cancer	HT	21	Response
Patient 2	Prostate cancer	C	17.5	Non-response
Patient 3	Breast cancer	C, RT	36.5	Non-response
Patient 4	Breast cancer	APD, HT	21	Response
Patient 5	Breast cancer	APD, C	10.5	Response
Patient 6	Prostate cancer	C	24.5	Non-response
Patient 7	Prostate cancer	C, RT, NTT	38	Response
Patient 8	Prostate cancer	C	12	Response
Patient 9	Prostate cancer	NTT	14.5	Response
Patient 10	Prostate cancer	C	21	Response
Patient 11	Prostate cancer	NTT	25	Non-response

*APD = Bisphosphonates, C = Chemotherapy, HT = hormone therapy, NTT = Novel Targeted Therapy, RT = radiotherapy.

Imaging was performed before and at a mean of 22 weeks (range 12 to 38 weeks) after initiating treatment. All patients were followed up clinically and radiologically for at least 12 months following the second MRI and were characterized as responders or non-responders based on combined clinical and radiological follow-up assessment (including PSA levels for prostate cancer patients, CT, anatomical MRI, SS and/or ^18^FDG-PET).

As there is no acknowledged gold standard for assessing treatment response of bone metastases, we adopted a reference standard based on all available imaging, clinical assessment and laboratory results available for each patient for at least 12 months [12]. Prostate Cancer Working Group Criteria 2 were used for patients with prostate cancer [13]. This assessment was made by two independent radiologists (NT and AP), not involved with the image analysis, in the setting of a tumor board, where the cases were discussed in detail in the presence of expert medical oncologists and radiation oncologists, and all clinical and radiological information were reviewed in detail. A patient was deemed to be a responder if the serial radiological tests showed improvement or absence of deterioration, supported by improvement in clinical symptoms (e.g. pain score) and/or improvement in laboratory markers (e.g. serum PSA, CA15-3 or circulating tumor cells). A patient was deemed a non-responder if there was radiological deterioration (including new adverse skeletal events and new bone lesions), increasing pain symptoms and/or worsening laboratory markers.

### MRI technique

WBDWI studies were performed on1.5T MR imaging systems (Avanto and Aera systems, Siemens Healthcare, Erlangen, Germany). Axial images were acquired using free breathing echo-planar diffusion-weighted imaging from the skull base to the mid-thigh level, sequentially across three to six imaging stations, each consisting of 50-30 slices respectively. At each imaging station, images were acquired using the following parameters: repetition time (TR) = 9000–14800 ms, echo time (TE) = 67–75 ms, matrix size = 128×128–150×150, slice thickness = 5–6 mm, receiver bandwidth = 1628–1961 Hz/pixel, 4–6 signal averages, STIR fat suppression with an inversion time (TI) of 180 ms, imaging field of view = 400±30 mm^2^ depending on patient size. Images were acquired using low/high b-value pairs of b = 50 and 900 s/mm^2^ for all 22 studies (11 pre and 11 post-treatment scans). Imaging protocols have been optimized independently for each scanner using the techniques discussed in [14] to reduce the magnitude of geometric distortions inherent to DWI, whilst maintaining high signal-to-noise ratio (SNR) and voxel resolution and obtaining global fat suppression. The current imaging protocol has been informed by a previous study where an SNR of approximately 22 (b = 50 s/mm^2^) and 15 (b = 900 s/mm^2^) were obtained using a similar imaging protocol on the same scanner within regions of prostate cancer metastases in lumbar vertebrae (unpublished data). In addition to WBDWI, anatomical imaging were also acquired using breath-hold axial T1-weighted fast low-angle shot gradient-echo (FLASH) imaging and/or coronal DIXON T1-weighted volume interpolated breath-hold gradient-echo (VIBE) imaging.

### Image processing and disease segmentation

**Figure 1:**
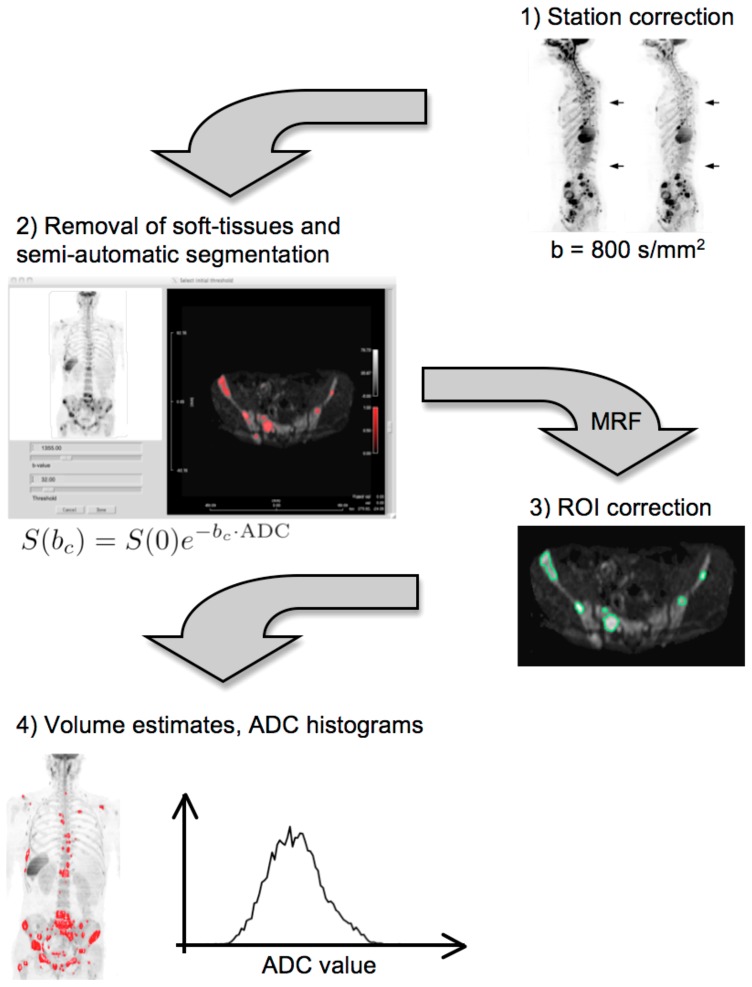
A flow diagram demonstrating the steps required for producing whole body volumetric tumor burden estimates and ADC histograms from WBDWI data. In step 2, S(0) and ADC represent estimates of signal intensity at b = 0 and the apparent diffusion coefficient respectively, calculated from acquired data. (MRF = ‘Markov random field’).

Image processing was performed using a proprietary IDL-based software (Exelis Visual Information Solutions, Inc.) by an experienced post-doctoral research fellow (MB) under the supervision of an expert radiologist, with eight years' experience in body DWI (DMK). Both reviewers were blinded to patient outcome during image processing. An outline of the segmentation procedure is presented in **Figure 1**:

**Step 1**. A post-processing algorithm is applied to the data to correct for spatial and intensity misregistrations between imaging stations [15]. The algorithm linearly scales the signal intensity of each imaging station so that the intensity profiles of images on either side of the station boundary have matched cumulative histograms. A shift along the phase-encoding direction is then applied to each station so that images on either side of the boundary are registered according to the minimum of the mean-square-difference of image intensities. This algorithm is applied in turn for each station.

**Figure 2:**
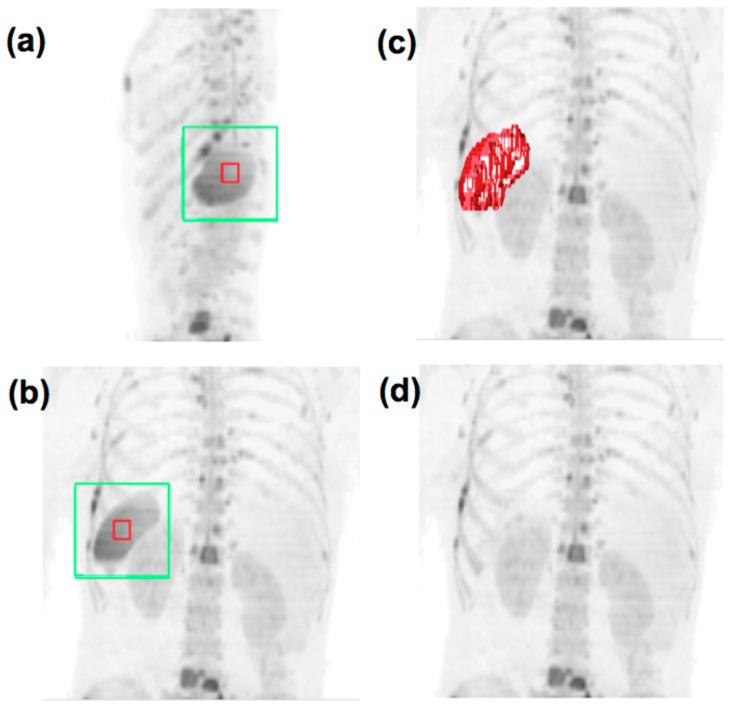
Coronal (a) and sagittal (b) MIP images of a patient with diffuse bone metastases from primary breast cancer shown at a computed b-value of 1355 s/mm^2^, with the selection box placed over the spleen. Voxels within the bounds of the red box are chosen as foreground seed points for the GrowCut algorithm whereas voxels on the surface of the green box are chosen as background seeds. Image (**c**) displays the results of the GrowCut algorithm as a red surface and (**d**) displays the data after removal of the spleen. In this way, the expert radiologist was able to refine VOI definitions by removing incorrect tumor segmentations.

**Figure 3:**
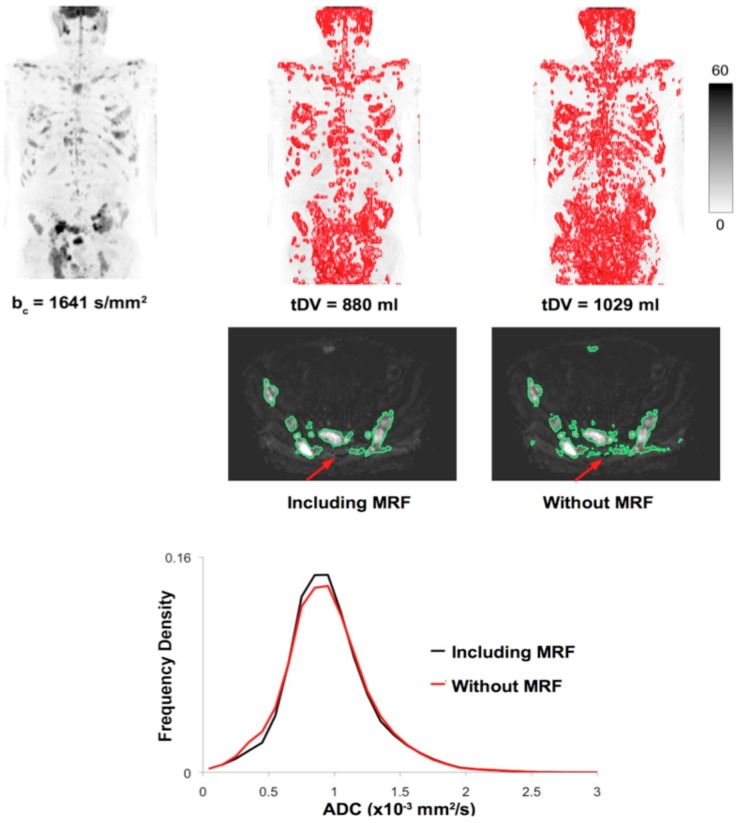
Comparison of segmentation results, achieved by simple thresholding and with the inclusion of a Markov random field (MRF) model, for a WBDWI data set of a patient diagnosed with multiple myeloma. It is clear that background noise and residual fat signal can be included in data that are only thresholded and that the MRF model can help to remove these spurious regions (red arrows). Although the resultant ADC histograms derived within these regions do not differ a great deal, simple thresholding results in an overestimate of tumour burden by 15%.

**Step 2**. The corrected data are processed using the cDWI technique to visually maximize the contrast between diseased and normal tissues by manually tuning the computed b-value using a sliding scale provided to the user during data visualization. A threshold is then manually selected, which maximizes visual suppression of signal from normal/benign tissue without suppressing signal from diseased areas. Residual signal from normal structures or artifacts is then removed using a three dimensional GrowCut algorithm developed by Vezhnevets and Konouchine (**Figure 2**), which uses cellular automaton as an image model to capture regions sharing similar statistical properties to the regions that are roughly initially identified by a user [11]. A Markov random field (MRF) model is applied to probabilistically smooth the classification results (details given in **Appendix S1**). A comparison of segmentation results achieved with and without the application of a MRF model is demonstrated in **Figure 3**. Applying the MRF model results in a smoothing operation, which reduces the number of voxels misclassified as disease in the presence of noise and incomplete fat suppression, compared with segmentation by simple thresholding of the WBDWI signal intensity.

**Step 3**.The segmentation process generates multiple regions of interests (ROIs) that are summed to produce volumes of interest (VOIs) of disease across the body. These can be viewed by maximum intensity projection (MIP) or multi-planar reformat (MPR). To avoid the inclusion of the brain, normal cervical nodes and salivary glands, data above the level of C4 were excluded from analysis. The expert radiologist further fine-tunes the segmentation by reviewing and manually excluding VOIs that are deemed erroneous (see next section) and the results are saved.

**Step 4**. The generated VOIs are used to calculate an estimate for the total body tumor burden of MR visible disease (in milliliters). These VOIs are transferred onto the corresponding ADC maps to derive gADC values. The distribution of ADC values across all lesions is visualized by histogram analysis.

The total processing time for a typical WBDWI data set consisting of 150×150×200 voxels is of the order of 30 minutes.

### Utility of WBDWI for estimating tumor burden and ADCs

Pre- and post-treatment estimates of tDV and distributions of gADC were derived for all patients. Applying the same computed b-value used for the pre-treatment data, VOIs for post treatment imaging were obtained and estimates of tDV and gADC were recorded.

The percentage change in tDV after treatment was compared between responding and non-responding patient groups. We also compared the absolute changes in gADC histogram parameters (median, variance, skewness and kurtosis) calculated before and after treatment by applying the same response categorization. We report on the median rather than the mean values of gADC due to their insensitivity to outliers and our previous observations that whole-body gADC histograms tend to be positively skewed [16].

### Statistical consideration

We performed a Mann-Whitney test to compare the percentage changes in tDV and changes in gADC histogram parameters between responding and non-responding groups of patients. For median gADC estimates, a one-tailed test was used due to the standard assumption that only increased ADC values are expected following successful treatment [3]. For all other calculated parameters, no such prior knowledge is available and two-tailed tests were used. A p-value of ≤0.05 was considered to be statistically significant.

## Results

The semi-automatic segmentation process was applied in all 11 patients, before and after treatment. However, in one patient, chemical shift and ghosting artifacts in the pelvis resulted in a substantial number of erroneous VOIs, which had to be manually removed following review by the radiologist.

**Figure 4:**
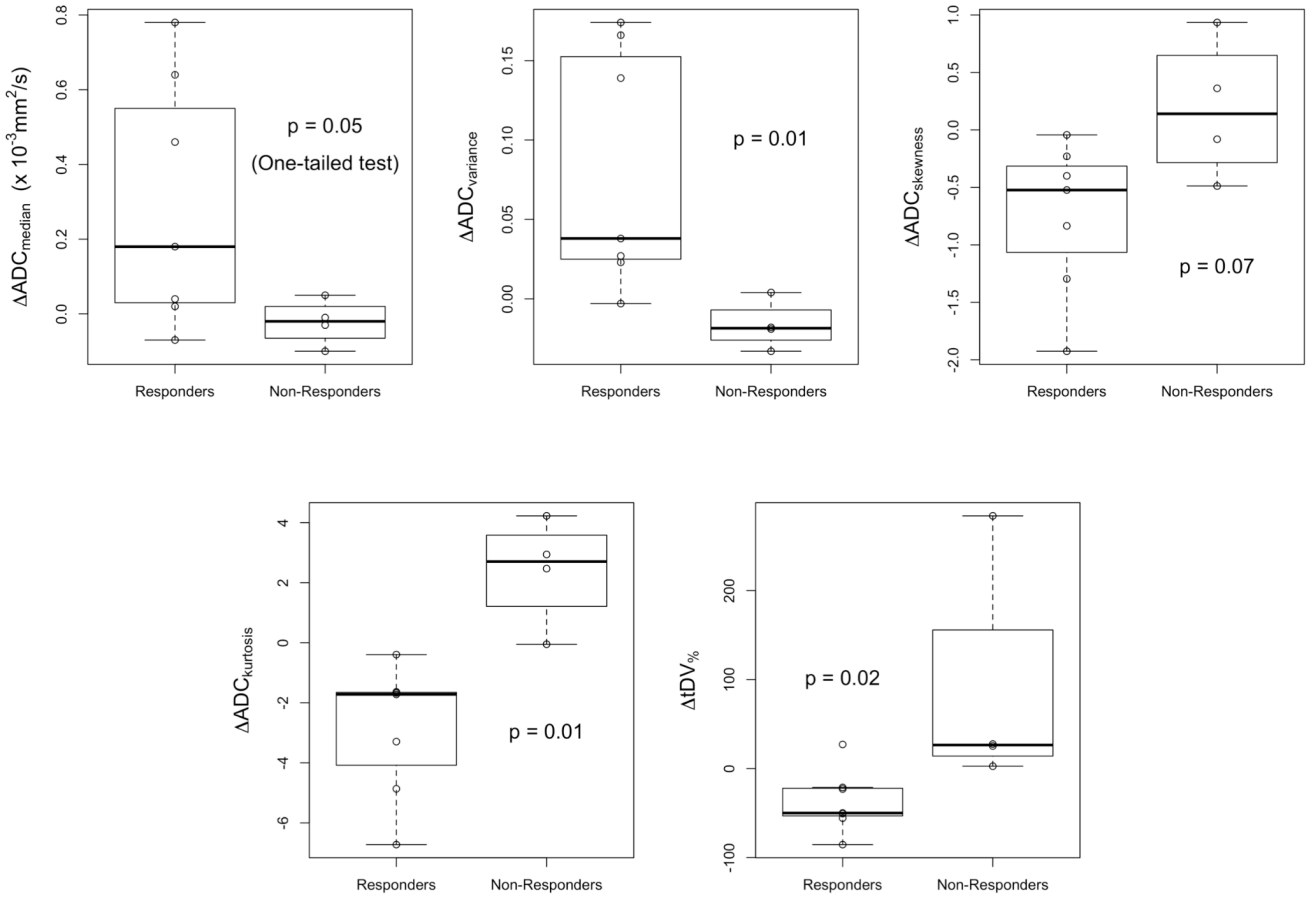
Boxplots of the percentage change in tDV and changes in gADC parameters after treatment in both patient cohorts. Results of the Mann-Whitney U test are displayed as p-values on each plot.

**Figure 5:**
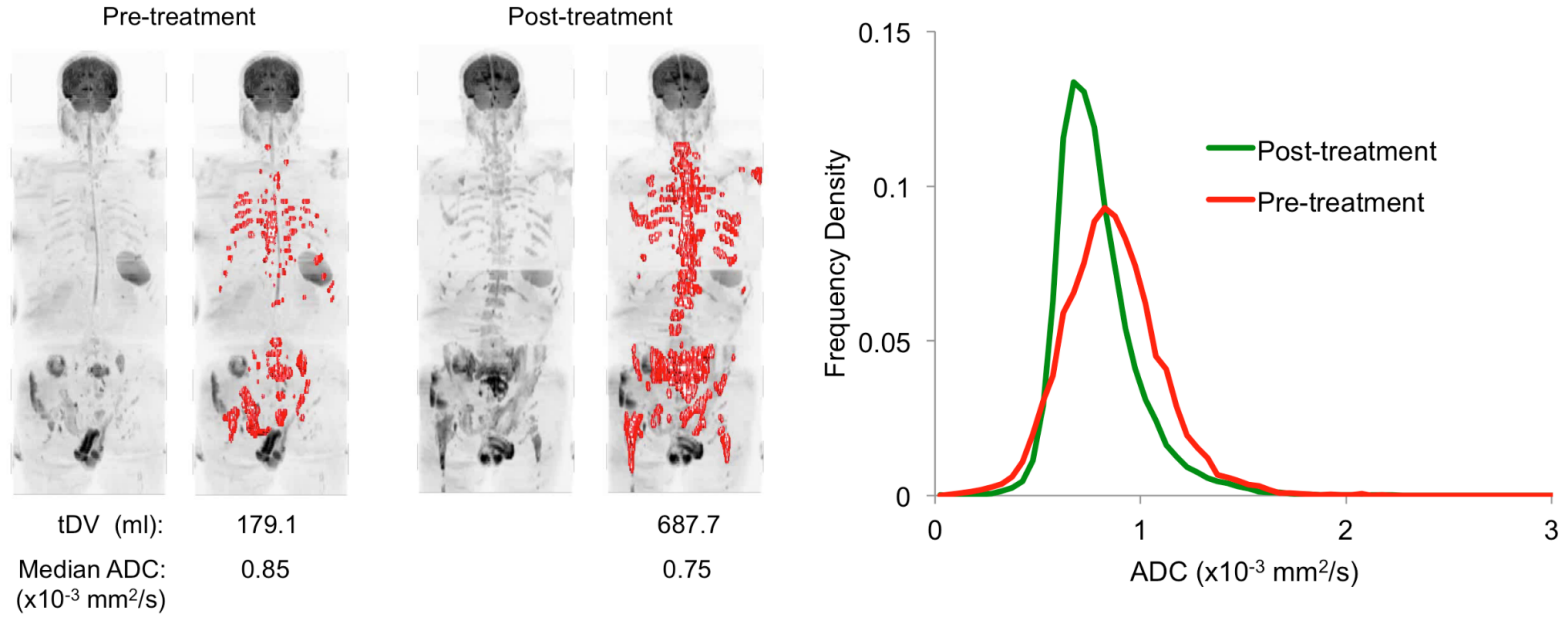
Treatment non-responder. The left figure demonstrates tumor burden estimates for a 78 year old male patient with metastatic prostate cancer (patient 6) displayed as a red surface on maximum intensity projection images before and after treatment (b = 900 s/mm^2^). A clear increase in tDV is observed after treatment for this patient. Images are displayed using the same windowing settings before and after treatment. On the right, ADC histogram distributions derived from the segmentation demonstrate a global decrease in ADC after treatment.

**Figure 6:**
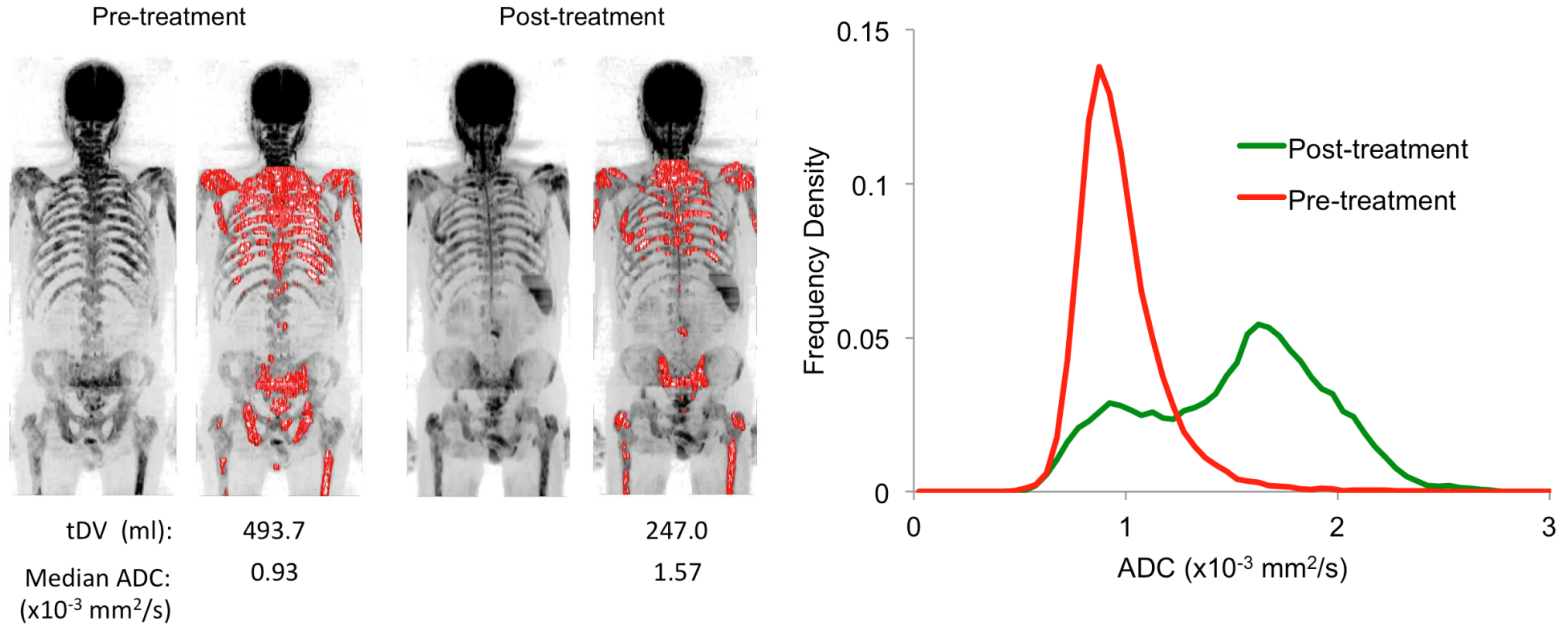
Treatment responder. The left figure demonstrates tumor burden estimates for a 71 year old male patient with metastatic prostate cancer (patient 10) displayed as a red surface on maximum intensity projection images before and after treatment (b = 900 s/mm^2^). A decrease in the estimated tDV is observed after treatment for this patient. Images are displayed using the same windowing settings before and after treatment. On the right, ADC distributions derived from the segmentation results shows significant increase in the median global value, with a clear shift of the histogram distribution to the right, indicating treatment response.

Boxplots of changes in gADC distribution parameters and percentage changes in tDV are presented in **Figure 4**. Patients classified as not responding to treatment showed a significantly larger percentage increase in tDV after treatment compared with responders (p = 0.02, Mann-Whitney test), whereas those patients classified as responding showed a significantly larger increase in median gADC (p = 0.05, one-tailed Mann-Whitney test). Of the other gADC histogram parameters calculated, a larger increase in gADC variance was observed for responding patients compared to non-responders (p = 0.01) whereas kurtosis was shown to decrease for responding patients and increase in non-responding patients (p = 0.01). Example tumor total volume images and full ADC distributions for Patients 1 (non-responder) and 2 (responder) are shown in **Figure 5** and **Figure 6** respectively.

Median values across responders are shown in **Table 2** for each of the derived parameters and in **Table 3** for non-responders. Among the responders, there was a significant percentage decrease in tDV, an increase in median gADC and gADC variance; as well as decrease in gADC skewness and kurtosis (p<0.05, Wilcoxon signed rank test). The population size for non-responders was too small to perform significance testing in that group.

**Table 2:** Median values of tDV and gADC distribution parameters across responding (N = 7) patient cohorts, before and after treatment administration.

Responders	tDV (ml)	gADC (median)	gADC (variance)	gADC (skewness)	gADC (kurtosis)
Before Treatment	137.4 (100.5–266.4)	0.82 (0.78–0.85)	0.03 (0.02–0.03)	0.81 (0.65–1.30)	2.30 (1.58–3.64)
After Treatment	79.7* (66.0–139.8)	0.91* (0.83–1.43)	0.06* (0.05–0.19)	0.08* (−0.15–0.45)	−0.38* (−0.64–−0.05)

Inter-quartile ranges for each value are shown in parentheses.

*Significant changes in values among responders before and after treatment are indicated (p<0.05, Wilcoxon paired rank-sum test: one-tailed test for median ADC, two-tailed test for all other metrics).

**Table 3:** Median values of tDV and gADC distribution parameters across non-responding (N = 4) patient cohorts, before and after treatment administration.

Non-Responders	tDV (ml)	gADC (median)	gADC (variance)	gADC (skewness)	gADC (kurtosis)
Before Treatment	207.0 (184.3–237.4)	0.81 (0.74–0.85)	0.06 (0.05–0.07)	0.58 (0.54–1.30)	1.37 (1.34–5.39)
After Treatment	286.3 (233.8–425.9)	0.78 (0.73–0.82)	0.03 (0.02–0.05)	0.99 (0.41–2.02)	4.02 (3.13–8.64)

Inter-quartile ranges for each value are shown in parentheses. Significance testing was not performed among non-responders because of small numbers within the cohort.

## Discussion

In this technical development report we describe a novel semi-automatic technique using Markov random fields for the segmentation of bone metastases from whole body diffusion-weighted MRI data. We have shown the feasibility of using this technique on a small pilot cohort of eleven patients with bone metastases arising from a range of primary malignancies; and derived estimates of tumor total diffusion volume (tDV) and tumor global ADC (gADC) before and after chemotherapy treatment. We found significant differences in the changes in tDV and gADC after treatment between the two groups of patients classified as responders or non-responders to treatment. Non-responding patients showed a significantly greater increase in tDV after treatment (p = 0.02) compared with the responding group. Responding patients showed a significantly larger increase in median gADC after treatment (p = 0.05). Furthermore, derivation of gADC histograms provides other statistical parameters such as the variance, skewness and kurtosis of the distribution. Although the utility of these metrics for characterizing disease and defining treatment response is still evolving, it is conceivable that these metrics may provide methods for quantifying heterogeneous response. Thus, our method shows substantial promise for evaluating metastatic bone disease, and may have a significant impact in drug development and clinical practice, where no standardized or reliable method of quantifying total burden or of evaluating the response of bony metastases to treatment has been established.

A number of potential limitations of our study should be mentioned. First, the segmentation technique is sensitive to the quality of the acquired DWI data, indicating the need for meticulous technique at image acquisition to minimize ghosting and other artifacts, which would result in erroneous VOIs due to the artifacts. The reviewing radiologist could manually remove these but this can be time consuming. Second, in all analyses, segmentation results above the level of the C4 vertebra were removed in order to eliminate potential spurious segmentation from normal soft tissues in the head and neck, such as the brain, salivary glands and lymph nodes. Whilst this may exclude some areas of disease, these do not have a major impact on the assessment of body burden of metastatic bone disease. Furthermore, except for a few select cases, disease was not observed in this region and it is more likely that false positives may arise here. Third, The utility of ADC and tumor burden estimates was tested only on a small cohort of eleven patients. Thus, it was not possible to obtain reliable estimates of the true diagnostic performance for tDV and gADC and the true potential of the technique is yet to be determined. Furthermore, the lack of a gold standard for measuring response of bone metastases makes interpretation of the true accuracy of the described technique difficult to define. In this pilot study, we have used all available clinical information for each patient to classify patients as either responding to treatment or not, using independent observers for this categorization to avoid bias. Fourth, we did not evaluate the interobserver variability of the technique in this initial analysis. However, the interobserver variability of this approach is currently being evaluated and if found to be reliable, will add weight to its utility in future trials. Last but not least the study was carried out on a small and heterogeneous patient population with differences in timings between pre- and post-treatment scans, which may impact on the sensitivity of technique for detecting post-treatment changes. In future trials with larger, more homogeneous patient populations it will be necessary to evaluate sensitivity of technique in relation to timing of the imaging and investigate the impact of our methods in specific tumor subtypes, corroborating these with a panel of clinical, laboratory and imaging tests. It will also be necessary to explore more robust segmentation strategies that provide a faster computation time, as current processing requires roughly 30 minutes, which may be regarded as being too long to be used clinically. One approach could involve segmentation of the entire skeleton followed by classification of disease based on ADC value, which could improve repeatability and require less user input.

## Supporting Information

Appendix S1 **Markov Random Field image classification.**
(DOCX)
