# Supplementary material for: Assessment of Treatment Response by Total Tumor Volume and Global Apparent Diffusion Coefficient Using Diffusion-Weighted MRI in Patients with Metastatic Bone Disease: A Feasibility Study
Source: PLoS One. 2014 Apr 7;9(4):e91779. doi: 10.1371/journal.pone.0091779 (PMC3977851; doi:10.1371/journal.pone.0091779)
Supplement: Appendix S1 — Markov Random Field image classification. (DOCX) [file pone.0091779.s001.docx]

**Appendix S1 – Markov Random Field image classification**

*A.1 Problem formulation*

Let $\mathbf{y}=y_{i}$ represent the cDWI data intensity of each voxel *i* within an acquired volume. To allow disease segmentation, it is necessary to classify each voxel into one of two classes: foreground, *F* (disease) or background, *B*. Let $\mathbf{x}=x_{i}$ represent the class of each voxel $i\in\{1,...,N\}$ such that $x_{i}\in\{F,B\}$. The problem of segmentation then involves specification of the probability, the ‘posterior’, of a set of voxel classes $\mathbf{x}$ given the cDWI data $\mathbf{y}$ that can be maximized to give a segmentation estimate. Using a Bayesian framework this may be written into the following form:

$$P(\mathbf{x}|\mathbf{y})\propto P(\mathbf{y}|\mathbf{x})P(\mathbf{x})$$

$P(\mathbf{y}|\mathbf{x})$ is the ‘likelihood’ of the data $\mathbf{y}$ given the classification$\mathbf{x}$, and $P(\mathbf{x})$ is the ‘prior’ distribution of the classification. In this paper we specify mathematical forms for $P(\mathbf{y}|\mathbf{x})$ and $P(\mathbf{x})$, which determine the classification posterior.

*A.2 Prior distribution,* $P(\mathbf{x})$

In this paper, models known as ‘Markov random fields’ (MRF) are used to define the prior distribution. These distributions are widely used in image analysis to model correlations between neighboring pixels, and provide the attractive feature that final segmentation results are probabilistically smoothed. The classification of any given voxel as disease or background is influenced both by its image intensity, where the degree of influence may be parameterized by scalar $\alpha$, and the majority vote of its neighbors, parameterized by scalar $\beta$. In this way isolated voxels are unlikely unless the image intensity provides significant evidence to suggest otherwise. To provide compromise between three-dimensional neighborhoods and increased computation time, we use a second-order neighborhood (8-neighborhood) in the axial image plane, and a first-order neighborhood (4-neighborhood) in the slice direction, which has relatively low spatial resolution. Furthermore, the influence of each neighboring voxel is scaled by the inverse of its Euclidean distance from the voxel in question so voxels that are further away have smaller influence in classification. Details of estimation values for $\alpha$ and $\beta$ are given below.

*A.3 Likelihood distribution,* $P(\mathbf{y}|\mathbf{x})$

The likelihood distribution is used to account for the influence of image noise on the classification scheme. It is usual to assume that given a labeling $\mathbf{x}$, the likelihood is independent at all sites *i* so that:

$$P(\mathbf{y}|\mathbf{x})=\prod_{i=1}^{N} p(y_{i}|x_{i})$$

In our segmentation methodology, it is necessary to define two conditional likelihoods for the image intensity; one for background signal, $p(y_{i}|x_{i}=B)$, and another for foreground, $p(y_{i}|x_{i}=F)$. We have found that theoretically valid background signal distributions, such as the Rician distribution, are unable to adequately characterize measured background noise in cDWI and so we derive a distribution from a set of two candidate models; gamma and inverse gamma, using the Bayesian Information Criteria (BIC) for model comparison [[1](#_ENREF_1)], details below. For the foreground distribution, $p(y_{i}|x_{i}=F)$*,* we use a uniform distribution whose width covers the range of image intensities within the total imaging field-of-view, and whose magnitude is chosen such that the likelihood is normalized.

*A.4 Implementation*

A variety of computational procedures exist for determining the voxel labeling that provides the maximum *a posteriori* estimate of $P(\mathbf{x}|\mathbf{y})$ [[2](#_ENREF_2),[3](#_ENREF_3)]. Considering the large amount of data involved in WBDWI we have implemented the Iterated Conditional Modes (ICM) algorithm proposed by Besag [[3](#_ENREF_3)]. The proposed algorithm proceeds as follows;

1. Initialize $\mathbf{x}^{\dagger}$ with labels determined from a user-supplied threshold, *t* (described in 'Material and Methods').
2. Select the model for background cDWI signal, $p(y_{i}|x_{i}=B)$, as the model with greatest BIC value for the voxels currently labeled as background.
3. Select $\alpha*$, such that at $y_{i}=t$:

$p(x_{i}=F|\alpha*)p(y_{i}|x_{i}=F)=p(x_{i}=B|\alpha*)p(y_{i}|x_{i}=B)$

This matches the foreground and background posterior probabilities at the user-defined threshold when the neighborhood interaction is not included.

1. Determine optimal random field parameter$\beta*$, by maximizing the pseudo-likelihood estimator (2), where $\partial i$ represents the neighbors of voxel *i*:

$$\beta*=\mathop{\mathrm{argmax}}\limits_{\beta} \prod_{i=1}^{N} p(x_{i}|x_{\partial i},\beta)$$

1. For each voxel *i* determine the classification, $x_{i}$, based on maximizing the conditional distribution $p(y_{i}|x_{i})p(x_{i}|\alpha*)p(x_{i}|x_{\partial i},\beta*)$ to create a new labeling estimate $\mathbf{x}'$.
2. If $\mathbf{x}'\neq\mathbf{x}^{\dagger}$ then set $\mathbf{x}^{\dagger}=\mathbf{x}'$ and return to (1), else stop the loop.

To limit processing time, we placed a limit on the number of loops taken to ensure that the algorithm stops and have found that good convergence occurs within <6 loops. For sites that are on the edge of the volume the neighborhood is adjusted accordingly to only include those neighboring sites that are within the volume.

1. Kass RE, Wasserman L (1995) A Reference Bayesian Test for Nested Hypotheses and Its Relationship to the Schwarz Criterion. Journal of the American Statistical Association 90: 928-934.

2. Geman S, Geman D (1984) Stochastic Relaxation, Gibbs Distributions, and the Bayesian Restoration of Images. Ieee Transactions on Pattern Analysis and Machine Intelligence 6: 721-741.

3. Besag J (1986) On the Statistical-Analysis of Dirty Pictures. Journal of the Royal Statistical Society Series B-Methodological 48: 259-302.
